# Performance evaluation of e-commerce firms in China: Using three-stage data envelopment analysis and the Malmquist productivity index

**DOI:** 10.1371/journal.pone.0255851

**Published:** 2021-08-11

**Authors:** Sufang Zheng, Rabnawaz Khan

**Affiliations:** School of Internet Economics and Business, Fujian University of Technology, Fuzhou City, Fujian Province, China; Szechenyi Istvan University: Szechenyi Istvan Egyetem, HUNGARY

## Abstract

As a new business form of international trade and electronic commerce, e-commerce has been a controversial topic that has attracted the attention of scholars and industry professionals. This study estimated the operating efficiency and total factor productivity (TFP) of listed e-commerce firms in China from 2015 to 2019. Three related methodologies were applied: data envelopment analysis (DEA), the Malmquist TFP index, and stochastic frontier analysis. The DEA analysis results showed that environmental variables exerted a substantial effect on technical efficiency. Most firms demonstrated effective technical efficiency after adjustment for input variables. Business-to-business firms had the highest operating efficiency, followed by business-to-consumer and production-to-consumer firms. Technical progress and scale were identified as two major factors affecting improvement in TFP. Hence, e-commerce firms should make full use of advanced technology and aim to achieve economies of scale.

## 1. Introduction

Advancements in information and communication technology can considerably improve infrastructure and enhance business efficiency. An increasing number of transactions are performed online. According to reports by Grand View Research, Inc., the global business-to-business (B2B) and business-to-consumer (B2C) markets were valued at US$5.7 trillion and US$3.35 trillion in 2019, respectively, and both B2B and B2C markets are expected to expand at compound annual growth rates of 17.5% and 7.9%, respectively, from 2020 to 2027 [1, 2]. Statista (2019) reported that the global market value of B2B e-commerce in 2019 was US$12.2 trillion, which is six times greater than that of B2C e-commerce [3]. Although these surveys have reported different market size calculations, they have indicated that B2B sales exceed B2C sales and that both are still growing.

This study adopts the Chinese e-commerce industry as the research object because under the condition of decreased foreign trade, e-commerce as a new business form has been thriving, accounting for an increased total Chinese trade volume. In 2019, import and export trade in e-commerce retail was US$26.6 billion, which is five times higher than that in 2015, with an annual growth of 49.5% [3]. This finding demonstrates the increasingly vital role of e-commerce in the Chinese economy.

E-commerce is becoming a new carrier for promoting industrial structure transformation and innovation-driven development as well as a new engine for facilitating economic growth. The use of new information technologies, such as cloud computing, big data, and artificial intelligence, has tremendously increased due to the blowout growth of the Internet economy. According to the China E-commerce Report 2019 published by the Department of E-commerce under the Ministry of Commerce, the trade volume of Chinese e-commerce in 2019 was valued at US$4973 billion, of which US$1518.5 billion were contributed by online retail trade sales, and the number of e-commerce practitioners was 51.26 million [4]. In 2019, global economic growth was slowing down, and risks and uncertainties in globalization and trade liberalization were increasing. By overcoming numerous difficulties and challenges, the Chinese economy is continuing to move ahead on the path of high-quality development. The digital economy, represented by e-commerce, has shown substantial progress and is playing a vital role in promoting domestic economic and social development.

From the perspective of the domestic market, Internet retail sales accounted for 45.6% of the total retail sales of social consumer goods in 2019 [5]. E-commerce has positively contributed to promoting consumption, stabilizing foreign trade, helping in poverty relief, and expanding employment, thus becoming a crucial factor responsible for stabilizing economic growth and promoting high-quality development. From the perspective of the international market, China has developed a bilateral e-commerce cooperation mechanism with 22 countries [6].

To our knowledge, few studies have used both three-stage DEA and DEA–Malmquist models to examine the e-commerce industry [7]. These studies have focused either on static analysis using the DEA–BCC model or on dynamic analysis using the DEA–Malmquist model. No study has combined the two models to evaluate overall efficiency.

Static analysis using the DEA model can reveal the relative efficiency and the reason underlying the improvement in efficiency after elimination of the effects of environmental factors. However, dynamic analysis is crucial because it can estimate the trends of changes in technological progress and technical efficiency. A change in technical efficiency is caused by a change in technological progress or technical efficiency, and a change in technical efficiency is caused by a change in pure technical efficiency or scale efficiency. In this study, the DEA–Malmquist index was used to solve the aforementioned problems.

In this study, we used DEA and the Malmquist productivity index (MPI) to examine the operation efficiency of e-commerce enterprises. Moreover, we classified 65 e-commerce enterprises into B2B, B2C, and production-to-consumer (P2C) categories and then separately analyzed the operations of the enterprises in these three categories by using data from the Tonghuashun database [8]. Previous studies have chosen sample firms either from the Chinese A-share market or the National Share Transfer System for small- and medium-sized enterprises. In this study, we included the top 66 listed e-commerce firms based on data from the China Electronic Commerce Research Center (CECRC) published in 2019. We selected input and output data from the Tonghuashun finance database, which is a public website widely used to obtain information regarding the Chinese stock market. Previous studies have usually classified e-commerce firms into three categories: platform based, application based, and service based [9]. However, we divided e-commerce firms into B2B, B2C, and P2C categories based on the field and industry they belonged to, as indicated by the data published by the CECRC.

This paper is divided into four parts. Section 2 provides a short literature review of the application of DEA and the Malmquist index in e-commerce and other areas. Section 3 describes the theoretical foundation and framework of DEA and the MPI and the process of selecting input and output indexes and introduces data collection resources. Section 4 provides details regarding the empirical analysis in this study, in which the three-stage DEA and MPI models were used to evaluate the operating efficiency and MPI of 65 e-commerce firms in China from 2015 to 2019; this section also provides the results. Finally, Section 5 offers some suggestions and measures to solve existing problems in e-commerce and presents the research conclusions.

This study estimated the operating efficiency and MPI of 65 e-commerce firms between 2015 and 2019 by using three-stage DEA, SFA, and DEA–Malmquist methodologies [10]. The DEA–BCC model was used to decompose technical efficiency into pure technical efficiency and scale efficiency. The MPI was employed to examine TFP and decompose this productivity change into changes in technology and technical efficiency [11]. The first-stage DEA measures the technical efficiency or operating efficiency before input adjustment based on original input data. The second-stage DEA involves performing a regression analysis of the effect of five environmental variables on input slack by using SFA. The third-stage DEA assesses the new technical efficiency after input adjustment and compares it with the findings obtained in the first-stage DEA. The goal of this study was to provide firms and industry professionals with some suggestions for improving their operating efficiency and performance.

## 2. Literature review

Under the ideological system of data envelopment analysis (DEA), an evaluation object is a decision-making unit (DMU) [12]. By calculating the rate of input and output of evaluation units, we can obtain the effective efficiency coefficient. Furthermore, this coefficient can be used to examine whether an evaluation unit is DEA effective as well as to understand the underlying reason for DEA effectiveness and identify means of improving DEA effectiveness using the projection method. Compared with traditional measurement methods, the main advantage of DEA is that it does not require the production function to be set in advance. Because of the indirect calculation of variable data in DEA, the nondimensionalization of data is required. Thus, DEA is superior because of its simplified computing and reduced errors.

Because DEA is a nonparametric method, it can prevent errors from occurring during the setting of parameters. DEA was developed by operational research experts they developed DEA and it is a multi-input and multi-output analysis method based on relative efficiency. Since the introduction of DEA in China by Wei, he has extensively applied in theoretical research [13]. Multi-input and multi-output indexes can be set to use DEA for calculating the technical efficiency, pure technical efficiency, and scale efficiency of any e-commerce enterprise to determine methods for improving the efficiency of businesses in the e-commerce industry. If the impetus for improving technical efficiency is pure technical efficiency, then the government should increase the level of Internet technology. If the impetus for improving technical efficiency is scale efficiency, then the government should promote business amalgamation.

The traditional three-stage DEA mode is constructed as follows: DEA–stochastic frontier analysis (SFA)–DEA [14–16]. In the first stage, an input-oriented BCC or CCR is applied to examine the efficiency of DMUs and input slack [17, 18]. In the second stage, the random cost mode in SFA is used to conduct regression analysis. The slack variable is decomposed into a set function, which is affected by three independent variables: environmental factors, random disturbance factors, and management inefficiency. In the third stage, after adjustment of the input, the adjusted input and original output variables are used to perform DEA again. DMU efficiency can be determined after elimination of the effect of the outside environment and random errors. The main deficiency of the traditional DEA model is in its second stage. Therefore, when DEA and SFA are combined to adjust the input, the slack is only a sum of the environment, random errors, and management inefficiency. Using only addition to adjust the original input and output is unreasonable. Second, when all DMUs are placed under the condition of the poorest environment and highest random disturbance, all DMUs eliminate the effect of the environment and random errors and encounter the same outside environment. This causes the problem of excessive adjustment of some of the inputs. This study focused on the effect of outside environmental factors on the input slack and defined environmental factors to examine their effects on different inputs. In addition, this paper proposes a new adjustment format on the basis of the estimation of environmental factors in the traditional SFA mode.

DEA–BCC and DEA–CCR methods can be used to calculate the relative effectiveness of various e-commerce firms; however, these methods cannot measure dynamic changes in the effectiveness of DMUs [17]. In 1994, Fare constructed a total factor productivity (TFP) change index based on the distance function [19]. To overcome the defects of the DEA model, Fare used two adjacent DMUs as a reference to compare and analyze the improvement in efficiency. The three-stage DEA is used to statically analyze technical efficiency, and the DEA–Malmquist index is used to dynamically analyze TFP [20]. Thus, the MPI can more comprehensively assess the overall efficiency. Moreover, this index can decompose the overall efficiency into efficiency change, technological progress change, pure technical efficiency change, and scale efficiency change and indicate their effect degree.

DEA is primarily used to calculate the operating efficiency of Internet companies, e-commerce firms, or businesses in other industries, such as listed firms in the food and beverage industry. Few studies have focused on e-commerce listed firms. Moreover, most studies have either used DEA or three-stage DEA to statically evaluate the operating efficiency of e-commerce firms or used only the DEA–Malmquist index to assess the trend of changes in TFP. He used DEA to estimate the production function for calculating the efficiency of Internet firms [20]. He included unique visitors and revenues as output variables and the number of employees, total operating expenses, and total assets as input variables. Furthermore, he divided e-businesses into three types: search, content, and e-tailer businesses. His results indicated that e-tailers were effective in gaining revenue, whereas search and content businesses were effective in attracting unique visitors. He applied DEA to assess the efficiency of e-commerce firms by including financial measures (profitability and capital utilization), operational measures (capacity and utilization), and e-commerce-specific measures (e-commerce site quality) as output variables and web technology investment, corporate operating cost, and the number of e-commerce staff as input variables [21]. On the basis of the results of his study, he suggested that managers should identify factors causing inefficiency and accordingly implement countermeasures. In addition, He used two-stage DEA to evaluate the performance of Internet companies and included the number of employees, total operating expenses, and total assets as input variables and the number of unique visitors and revenue as output variables [22]. The results indicated that the model could effectively evaluate operating efficiency. He used DEA and decision trees to analyze the efficiency and recommendation of B2C controls, and the results showed that the efficiency of retail firms and information service providers was more satisfactory than that of financial firms [23].

Some Chinese scholars have used DEA and other methods to estimate the operating performance of e-commerce firms. She applied two-stage DEA to assess the scale, congestion, efficiency, and effectiveness of e-commerce firms, and the results indicated that ineffective scale efficiency and the use of outdated technology caused inefficiency [24]. He used the three-stage DEA model to evaluate operating efficiency after eliminating the effects of environmental and random factors [25]. The results indicated that government subsidy and regional Internet development level were key factors for improving operational efficiency, whereas years of incorporation, degree of regional openness, and equity concentration were factors restricting the improvement of operational efficiency. He used the DEA–BCC model and super-efficiency model to empirically analyze the operating performance of e-commerce firms [26]. The results of that study revealed that 7 out of 14 firms had effective DEA, 11 firms had effective pure technical efficiency, and most firms with ineffective scale efficiency had increasing returns to scale.

A few researchers have applied the DEA–Malmquist index to calculate the efficiency of e-commerce firms. He used DEA and the MPI from a multi theoretical perspective to examine the TFP growth of IT service industries in OECD countries, and the results showed that technological progress was the key factor for the growth of productivity [27]. He used the MPI model to discuss the change in TFP by dividing firms into three categories: B2B, B2C, and OTA (Online Travel Agency) [7]. The results of the study indicated that technical change plays a crucial role in driving changes in TFP. She applied the DEA–BCC model and MPI to analyze technical efficiency statically and dynamically and discovered an increase in TFP [23]. Most firms had effective pure technical efficiency, and low technical efficiency could be attributed to low scale efficiency. Li also provided some suggestions, such as adopting fine management, advanced management styles, and operating cost control for improving technical efficiency.

Other methods have also been used to calculate the overall efficiency of e-commerce firms. He evaluated the overall efficiency of Chinese e-commerce firms by performing principal component analysis and cluster analysis on 48 listed firms from the Chinse A-stock market [28]. Firms were divided into three categories: third-party e-commerce platform firms, online transaction firms using a self-owned platform, and e-commerce service firms including third-party payment, logistics, delivery, and storehouse. The results indicated that the overall performance of the firms was low, the same firm exhibited fluctuating performance, and few firms showed balanced development. She constructed an evaluation index system and performed multiple linear regression to explore the production factor influencing the efficiency of e-commerce development [26]. E-commerce trade volume was selected as the dependent variable, and fixed asset investment and the number of college graduates were the independent variables; the control variables were the number of e-commerce firms, express income, Internet penetration rate, and number of netizens. In addition, They assessed the performance of e-commerce firms in Ningbo, Zhejiang Province using a technology-organization- environment (TOE) model [14]. They investigated the international competency of Chinese e-commerce logistics firms by using the analytical hierarchic process (AHP) method and proposed policies for improving international competency [29]. They estimated the operating performance of Chinese e-commerce by using the Delphi and AHP methods. They indicated the presence of substantial regional disparities in e-commerce operations and made some suggestions from the perspective of enterprise cultivation, talent introduction, and infrastructure construction. The aforementioned studies have laid the foundation for the present study’s selection of e-commerce operating indexes and development of an e-commerce operating performance model [19].

Silk Road e-commerce has become a new channel of trade cooperation and has promoted the digital economic development of partner countries, thus attracting their increasing attention. Although Chinese cross-border e-commerce has substantially flourished and technical efficiency has considerably improved in recent years, whether it is responsible for the scale expansion or technological progress and efficiency remains unclear; clarifying this matter was the goal of this research.

In summary, most studies have focused only on a specific area, such as financial performance or logistics efficiency for the examination of e-commerce. In the current study, panel data from 65 e-commerce firms were used to construct a DEA approach combined with the Malmquist TFP index to explore TFP and technical efficiency, and the CRS and VRS were used to decompose e-commerce. The findings of this study offer major contributions to the literature [30].

## 3. Data and methodology

### 3.1 DEA method

DEA can be divided into input oriented and output oriented [31]. Output-oriented DEA is used to achieve the maximum output under the condition of fixed input. Input-oriented DEA is used to reduce the input and achieve the optimal configuration under the condition of fixed output. Typical models of DEA are the CRS and VRS models. The CRS model is used to calculate overall efficiency under the assumption of fixed returns to scale. The VRS model is used to calculate the pure technical efficiency and scale efficiency of each DMU based on variable returns to scale by model 1 and 2. This study used both models and considered every year as an individual DMU to calculate overall efficiency, pure technical efficiency, and scale efficiency.

**Model 1**: **CRS model**

$$\begin{array}{l} \;\mathrm{Min}\theta_{c}\\ s.t\left\{ \begin{array}{c} \theta_{c}x_{0}-\sum_{j=1}^{N}\lambda_{j}x_{j}\geq 0\\ -y_{0}+\sum_{j=1}^{N}\lambda_{j}y_{j}\geq 0\\ \lambda_{j}\geq 0\\ j=1,2,\ldots ,N\\ \theta_{c}\geq 0 \end{array}\right. \end{array}$$ (1)

In Eq (1), x_0_ and y_0_ represent the input and output vector, respectively, for tea production. Furthermore, x_j_ and y_j_ represent the jth input and output vector of tea production, respectively. λ_j_ represents the weight of each DMU. θ_c_ represents overall efficiency under the condition of fixed returns to scale (value between 0 and 1), which reflects the extent of the input–output efficiency of tea production. If θ_c_ = 1, then the input–output is considered to be completely effective (i.e., high technical and scale efficiency). E-commerce businesses are considered to possess technical efficiency if they make full use of available resources to achieve the maximum output and best operational conditions, and they are considered to possess scale efficiency when their profit is at the stage of fixed returns to scale (i.e., the output increases or decreases with the same ratio as the input). If θ_c_ < 1, then the existing technical usage and the configuration of the production factor are considered to not be at the best condition.

**Model 2**: **VRS model**

$$\begin{array}{l} \mathrm{Min}\theta_{v}\\ \;s.t.\{\begin{array}{c} \theta_{v}x_{0}-\sum_{j=1}^{N}\lambda_{j}x_{j}\geq 0\\ -y_{0}+\sum_{j=1}^{N}\lambda_{j}y_{j}\geq 0\\ \lambda_{j}\geq 0\\ j=1,2,\ldots ,N \end{array} \end{array}$$ (2)

In Eq (2), θ_v_ represents pure technical efficiency (value ranging from 0 to 1) and can be used to calculate the extent to which pure technical efficiency causes overall inefficiency under the condition of input in e-commerce firms. The other variables have the same definitions as those used in the CRS model.

In Eq (3) the relationship of the overall efficiency θ_c_ with the hypothesis of fixed returns to scale with the scale efficiency θ_y_ under the condition of changeable returns to scale and the scale efficiency θ_s_ is expressed as follows:

$$\theta_{c}=\theta_{v}\times \theta_{s},\;\theta_{s}=\theta_{c}/\theta_{v}$$ (3)

The conversion relationship among overall efficiency, pure technical efficiency, and scale efficiency can be examined to measure the scale efficiency of each DMU θ_s_. It can be used to estimate whether the scale of e-commerce firms is optimal under the condition of fixed input.

### 3.2 Stochastic frontier approach (SFA)

In the second stage, the SFA random cost model was used to perform regression analysis. Slack variables were decomposed into collection functions, namely environmental, random-disturbance, and ineffective-management factors, expressed as follows:

$$S_{ni}=f^{n}(Z_{i};\hat{\beta_{n}}+V_{ni}+U_{ni})$$ (4)

where N = 1, 2, …. N represents n inputs, i = 1, 2, …, m represents the ith DMU, and S_ni_ Eq (4) is the nth slack variable of the ith DMU. $f^{n}(Z_{i};\hat{\beta_{n}})$ is the effect of environmental factors on slack variables. Z_i_ represents environmental factors. $\hat{\beta_{n}}$ is the coefficient of environmental factors. V_ni_ + U_ni_ is the mixed error term, V_ni_ represents the random error and normal distribution, U_ni_ represents ineffective management and partial normal distribution, and V_ni_ and U_ni_ are independent variables.

Regarding the separation formula of ineffective management, studies conducted in China have indicated that the formula has many forms, and considerable differences exist among the different forms. Drawing on his research and he argued that ineffective management could be separated into different categories [31]. However, he applied the production function, and its form of mixed error was ε = ν + μ. He used the cost function form, which significantly differs from that others. Thus, on the basis of the study conducted by ineffective management, the cost function used in this study is as follows:

$$E(\mu /\varepsilon )=\sigma *\left[\frac{\emptyset (\lambda \frac{\varepsilon }{\sigma })}{\Phi (\lambda \frac{\varepsilon }{\sigma })}\right]+\lambda \frac{\varepsilon }{\sigma }$$ (5)

In Eq (5) $\sigma *=\frac{{\sigma_{\mu }\sigma }_{\nu }}{\sigma }$, $\sigma =\sqrt{\sigma_{\mu +}^{2}\sigma_{\nu }^{2}}$, λ = σ_μ_/σ_ν_. ∅(·) and φ(·) respectively represent the density function and distribution function. Subsequently, the estimation formula of the random error μ can easily be obtained:

$$E(\frac{\nu_{\mathrm{ni}}}{\nu_{\mathrm{ni}}+\mu_{\mathrm{ni}}})=\mathrm{Sni}-f^{n}(\mathrm{Zi};\hat{\beta_{n}}-E(\mu_{\mathrm{ni}}/\nu_{\mathrm{ni}}+\mu_{\mathrm{ni}})$$ (6)

In Eq (7) the purpose of SFA regression analysis is to eliminate the effects of environmental and random factors on efficiency calculation so that all DMUs are adjusted under the same external environmental conditions. The adjustment formula is as follows:

$$X_{\mathrm{ni}}^{A}=X_{\mathrm{ni}}+[\max\;(\mathrm{fn}\;(\mathrm{Zi};\hat{\beta_{n}}))-\mathrm{fn}(\mathrm{Zi};\hat{\beta_{n}})]+[\max\;(\nu_{\mathrm{ni}})-\nu_{\mathrm{ni}}]$$ (7)

In Eq (7) $X_{ni}^{A}$ is the input after adjustment; X_ni_ is the input before adjustment; [$\max\;(f_{n}(Z_{i};\hat{\beta_{n}}))-fn(Zi;\hat{\beta_{n}})$] is the adjustment for the outside environmental factors. [max (ν_ni_)-ν_ni_] is used to put all DMUs in the same random condition.

In the third stage, after input adjustment, DEA is performed using the adjusted input and original output value; subsequently, DMU efficiency is calculated after elimination of the effect of environmental factors and random errors.

### 3.3 DEA–Malmquist index model

Charnes et al. [32] proposed the Malmquist index in 1953. It was originally used to calculate the trend of changes in the consumption index Until 1992–1994, he used the DEA method which is developed in 1978. The nonparametric linear programming algorithm of the Malmquist index [33]. Currently, the Malmquist index proposed and frequently used. Its expression is as follows:

$${\mathrm{MI}}_{0}=\left(x_{t+1},y_{t+1},x_{t},y_{t}\right)={\left[\frac{D_{0}^{t}(x_{t+1},y_{t+1})}{D_{0}^{t}(x_{t},y_{t})}\times \frac{D_{0}^{t+1}(x_{t+1},y_{t+1})}{D_{0}^{t}(x_{t},y_{t})}\right]}^{\frac{1}{2}}$$ (8)

In Eq (8) the expression, X_t + 1_, Y_t + 1_ and X_t_, Y_tm_ respectively describe the input and output vector quantity in the next and current period. $D_{1}^{t+1}$ and $D_{0}^{t}$ describe the technical efficiency in the next and current period with reference to the current period. TFPch = M_0_ (Xt, Y_t_, X_t + 1_, and Y_t + 1_) > 1 means that TFP is increasing; otherwise, it is declining.

According to Fare’s analysis results, under the hypothesis of constant returns to scale, TFP change (TFPch) consists of efficiency change (EC) and technical change (TC); that is, TFPch = TC × EC. TC > 1indicates that technical progress is improving, whereas TC < 1 indicates that technical progress is declining. EC > 1 indicates that technical efficiency is improving, whereas EC < 1 indicates that technical efficiency is declining. EC consists of pure technical efficiency change (PC) and scale efficiency change (SC); accordingly, the equation is EC = SC × PC. Subsequently, we have the equation TFPch = TC × SC × PC. Two methods can be used to calculate the MPI: the nonparametric DEA method and the parametric SFA method. SFA must assume the production function expression in advance and then estimate the parameter. Moreover, SFA can only deal with a single output and cannot suggest improvement measures. However, DEA does not require a hypothesis for the relationship between input and output and evaluates the relative effectiveness of production units. Furthermore, DEA can analyze the multi-input and multi-output and suggest measures for improving the target of every output and reducing the target of input to maximize efficiency. Thus, this study used the DEA method to calculate the MPI. MPI > 1 indicates that TFP is increasing; otherwise, it is considered to be decreasing. TC > 1 indicates that TC is the main factor responsible for the increase in TFP; otherwise, TC is considered to reduce TFP. The connotation of EC is equal to technical progress. The two factors affecting technical efficiency, namely SC and PC, reflect the effect degree. The higher the score, the greater the effect on technical efficiency.

### 3.4 Sample selection

This study divided e-commerce enterprises into three types: (1) Type I: B2B, wherein 30 party e-commerce platforms use the Internet or mobile communication to perform business activities such as product display, information publishing, and price guidance. They use service functions such as online brokers, online transactions, and online or mobile payments. (2) Type II: B2C, wherein e-commerce platforms conduct online transactions by using the third-party e-commerce platform or self-owned e-commerce platform. (3) Type III: P2C, wherein enterprises provide comprehensive and targeted service support based on hardware, software, and networks, including third-party payment, logistics, delivery, and storehouse [34].

The CECRC publishes the most authoritative economic data in the field of e-commerce and sends these data regularly to many ministries, such as the Ministry of Commerce and Ministry of Industry and Information, China Statistical Bureau, and hundreds of e-commerce associations [35]. Thus, it has become the most important data resource and is used for decision-making by thousands of e-commerce enterprises, electrical business parks, and listed companies nationally.

Regarding the concept of e-commerce enterprises, on the basis of the description of the main business and its percentage in the annual financial statement of listed companies, this study chose 65 e-commerce companies listed in the Chinese A-stock market and in Hong Kong and US stock markets. These 65 firms were included in the Analysis Report on Chinese E-Commerce Listed Companies Market Value Data 2019 in the top 66 firms published by the CECRC. However, one listed company, YOUXIN, did not publish financial statements in the past 5 years, its data were unavailable. All data were obtained from financial statements published on Tonghuashun, a popular financial website in China. Among these 65 firms, 17 were B2B firms, 27 were B2C firms, and 21 were P2C enterprises [36].

Until December 31, 2019, the total market value of these 66 e-commerce listed companies was US$921.4 billion, which increased by 63.31% compared with the market value at the end of 2018 (i.e., US$564.2 billion) [37]. Overall, the total market value of e-commerce listed companies was increasing, indicating that the market was optimistic regarding this new economic industry. Many companies experienced significant growth in market value. For instance, Vipshop, Metituan, and PingAn Health Cloud experienced a growth of 202.96%, 149.92%, and 109.76%, respectively. However, some companies experienced a significant decline in their market value; for instance, Mogujie, Sinocan, and Tuanche experienced a decline of 87.28%, 81.63%, and 80.68%, respectively.

The total market value of Chinese e-commerce in 2019 was US$921.7 billion, and the following six companies had a market value of more than US$1 billion: Alibaba, Meituan, Jingdong, Pinduoduo, Xiaomi Group, and Ctrip [24]. The market value of the following six companies ranged from US$7000 million to US$1 billion: Alibaba Health, Sunninig, 58.com, Vipshop, Luckin coffer, and Ping An Health Cloud. Furthermore, the following 16 companies had a market value ranging from US$1000 million to US$7000 million: 51job, Genshuixue, Alibaba Picture Group, Nanjing, Tongcheng-Elong, Three Squirrels, Lexin, Koolearn Technology, Maoyan, Baozun, Onechance, Gome, Shanghai Ganglian, GlobalTop, Youdao, and Guolian. The remaining 38 companies had a market value of <US$1000 million [38]. Generally, the total market value was growing, but polarization was observed. B2C and P2C companies had a higher market value, and B2B companies had a lower market value.

### 3.5 Variable selection

#### 3.5.1 Input and output variables

In the DEA method, input and output indexes that represent the operating management level of e-commerce firms must be selected. The choice of indexes should abide by the principle of objectivity, scientificity, comprehensiveness, and operability. On the basis of the aforementioned principle, this study developed an index system for evaluating the performance of e-commerce firms. The option of input and output factors objectively embodied the competency of evaluation and demonstrated management controllability; in addition, the availability of variable data was considered. In practice, in the DEA model, the total amount of the input and output index should not be more than half of the amount of DMUs. To achieve better results, there should be fewer input indicators and more output indicators [39].

**Fig 1:**
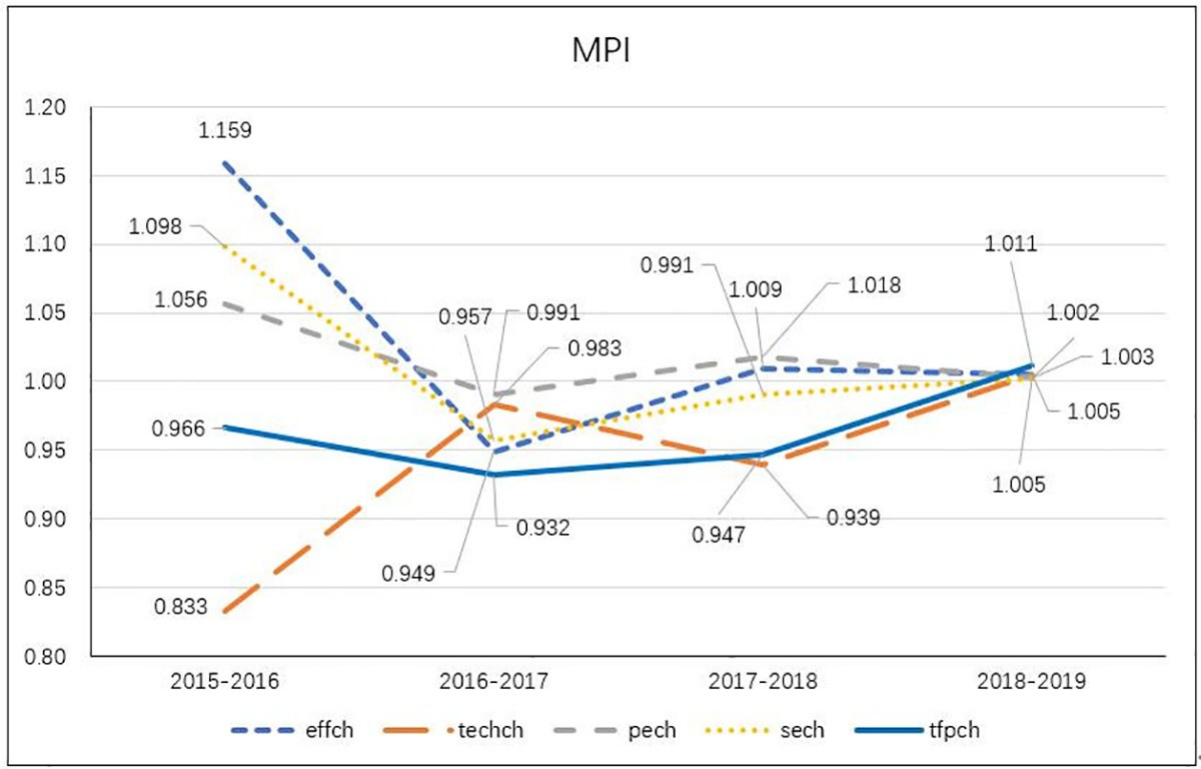
Summary of the Malmquist index of annual means.

On the basis of the aforementioned principle of input and output variables for the DEA model, this study chose three input indexes, namely the total assets (in RMB), corporate operating cost (in RMB), and number of employees, and two output indexes, namely corporate operating revenue (in RMB) and net profits (in RMB). Fig 1 shows the conceptual mode of e-commerce efficiency.

The total assets and number of employees are input variables, and they directly affect the economic benefits of enterprises. Operating cost is the cost expenditure of firms for selling products or providing services. Combined with operating revenues, operating cost can reflect the situation of the main operations of firms. E-commerce has the benefit of requiring a lower operating cost, especially for service firms that provide logistics, delivery, and transportation services. This provides a good opportunity for these firms to reduce their operating costs. Two outputs were selected: Net profits were chosen as an output because they can reflect the profitability of e-commerce companies. Operating revenues were chosen as an output because they represent the development potential for e-commerce companies.

#### 3.5.2 Environmental factors

1. To meet the requirement of the separation hypothesis, environmental factors should be uncontrollable factors that exert a considerable effect on the operating efficiency of firms [40]. This study combined the development characteristics of e-commerce with relative research results and chose the following five indexes as environmental variables Years of incorporation: With an increase in the number of years of incorporation, the management experience of enterprises gradually increases and the operating efficiency improves accordingly ownership concentration: Excessive ownership concentration is reported to cause an overlap between the interests of the whole enterprise and those of the first shareholder, resulting in the phenomenon of an “internal controller.” Shareholders with a controlling advantage may sacrifice the interests of the whole enterprise to earn for themselves, thereby causing operational inefficiency Equity restriction ratio: Reports have differed regarding the effect of the equity restriction ratio. Some scholars believe that the equity restriction ratio can improve the supervision capability and motives of minority shareholders, whereas other scholars have found that a high equity restriction ratio exerts a negative effect on the operation of enterprises.
2. Regional Internet development level: This factor is represented by the Internet development index. E-commerce cannot develop without improvement in infrastructure. The Internet is the catalyst of e-commerce development and growth. Netizens, who are rapidly growing in number worldwide, are the user basis for e-commerce development. The application of big data upgrades the information-matching and transaction-closure function, leading to changes in the business model. The resistance of the blockchain to being dominated can reduce business fraud and optimize the service experience of consumers. The data source of the Internet development index is the China Internet Report 2019, and this index is based on a comprehensive evaluation of provinces in terms of infrastructure construction, innovation capability, development of the digital economy, Internet application, network security, and network management [41]. Degree of regional opening up: An improvement in the degree of regional opening up can be helpful to the flow of elements. The absorption of information and technology during opening up contributes to the improvement of enterprise competency. Economic openness is the premise and basis for regional opening up. Regarding data availability, this study used the degree of foreign trade dependence to measure the degree of regional opening up. Data regarding regional import and export trade value and regional gross domestic product were obtained from the website of the National Bureau of Statistics.

### 3.6 Negative transformation

Some enterprises had negative profits. However, because negative values cannot be used in the DEA model, transformation was required. After reviewing the literature, we identified four methods for transforming negative values into positive values. The first method is nondimensionalization, which involves transforming the negative number into a positive number close to 0. The second method is writing the input and output vectors as matrices and performing elementary row transformation. The third method is to directly transform all negative numbers into positive numbers. The fourth method is to perform data translation by using the add-value transformation. Among these four methods, elementary transformation is universal and suitable for any negative output to obtain the correct efficiency. Hence, this study used the elementary transformation method by using (KJT)_ij_ × 1+Z _ij_ to transform negative values into positive values. As shown in Table 1, 8.49% of variables result is negative from 1625, which obtained from total dataset 65 firms [42].

**Table 1:** Positive and negative firm data.

No	Companies Name	Area	Industries	Types	Net profit				
					2015	2016	2017	2018	2019
1	Jingdong	Retail	Comprehensive	B2C	-	-	-	-	+
2	Pinduoduo				-	-	-	-	-
3	Xiaomi		3C		-	+	-	+	+
4	Three squirrels		Food		+	-	+	+	+
5	Lexin		Finance		-	-	+	+	+
6	Gome		Comprehensive		+	-	-	-	-
7	Qudian		Finance		-	+	+	+	+
8	Yunji		Membership		-	-	-	+	-
9	Weimob		Service		-	-	+	-	+
10	Youzan				-	-	_	_	-
11	Ruhnn		Internet Celebrity		-	-	+	+	-
12	111.com		Medicine		-	-	-	-	-
13	Babytree		Infant & Mon		-	-	-	+	-
14	Mogu		Fashion		-	-	-	-	-
15	Jumei				+	+	-	+	+
16	Secoo		Luxury		-	-	+	+	+
17	Tuanche		Motormobile		-	-	-	-	-
18	Shanghai Ganglian	Industry	Steel	B2B	-	+	+	+	+
19	United Information	Cross border e-commerce	Export		-	+	+	+	+
20	Guanfu	Industry	Plastify		+	+	+	-	+
21	Molbase				-	-	-	-	-
22	HC Group		Comprehensive		+				-
23	Europol				+	+	+	-	+
4	LightInTheBox	Cross border e-commerce	Export		-	-	+	+	+
25	DX.com				+	-	+	+	+
26	Meituan	Life Service	Comprehensive	P2C	-	-	-	-	+
27	Ctrip		Online Travel		+	-	+	+	+
28	Alibaba Health		Online Medicine		-	-	-	-	-
29	58.com		Life Information		-	-	+	+	+
30	Luckin		Catering takeout		+	-	-	-	-
31	Ping An Health Cloud		Online Medicine		-	-	-	-	-
32	51job		Internet Recruitment		+	+	+	+	+
33	Genshuixue		Online Education		-	-	-	-	+
34	Alibaba Picture		Internet Film and TV		+	-	-	-	-
35	Tongcheng-Elong		Online Travel		-	-	+	+	+
36	Koolearn		Online Education		+	+	+	+	+
37	Maoyan		Online Ticket		-	-	-	-	+
38	Youdao		Online Education		+	-	-	-	-
39	So-Young		Online Medical Beauty		+	-	+	+	+
40	Fangdd		Online Accommodation		-	+	+	+	+
41	Q & K				-	-	-	-	-
42	Qeeka		Internet Home Decoration		-	-	-	+	+
43	Tuniu		Online Travel		-	-	-	-	-
44	Leju		Online Accommodation		+	-	-	-	+
45	Soufun				-	-	+	-	-
46	51Talk		Online Education		-	-	-	-	-

## 4. Empirical results and discussions

### 4.1 First-stage DEA results

The original input and output data were analyzed by the DEA approach, and the results are presented in Table 2. In general, the operating efficiency of e-commerce industry exhibited a declining trend. Without consideration for environmental and random factors, the average operating efficiency of 65 e-commerce firms was calculated to be 0.773 in 2019, which was above the moderate level. The average scale efficiency and pure technical efficiency were 0.872 and 0.675, respectively. Before adjustment of the input variables, the ineffective pure technical efficiency of e-commerce firms resulted in their ineffective operating efficiency, indicating that some improvement occurred in resource allocation and operation management. Regarding different firm types, the technical efficiency of B2C firms was higher than that of B2B and P2C firms. B2B and P2C firms initially exhibited an increase and a subsequent decrease in technical efficiency, whereas B2B firms demonstrated a decrease and a subsequent increase in technical efficiency. In addition, we observed a significant difference in effective operating efficiency (TE = 1) among firms. In 2019, 11 firms (three B2C firms, five B2B firms, and two P2C firms) were observed to have effective operating efficiency. Furthermore, 32 and 15 firms had operating efficiencies of <0.7 and <0.5, respectively. Of the 65 firms, Fangd and Alibaba Picture had the lowest operating efficiency. The BCC model analysis based on original data.

**Table 2:** Technical efficiency, pure technical efficiency, and scale efficiency of 65 e-commerce firms under the BCC in 2019.

No.	DMU	crste	vrste	scale	return to scale
	B2C				
1	Alibaba	0.492	1	0.492	drs
2	Jingdong	0.820	1	0.820	drs
3	Pinduoduo	0.711	1	0.711	drs
4	Xiaomi Group	0.722	1	0.722	drs
5	Sunning	0.670	1	0.670	drs
6	Vipshop	0.809	1	0.809	drs
7	Nanji	0.891	0.904	0.986	irs
8	Three squirrels	0.896	0.933	0.960	drs
9	Lexin	0.673	0.754	0.893	drs
10	Baozun	0.818	0.895	0.913	drs
11	Onechance	0.741	0.757	0.980	drs
12	Gome	0.547	0.799	0.684	drs
13	Qudian	1	1	1	-
14	Bear	0.676	0.733	0.923	drs
15	Zhidemai	0.731	0.789	0.926	irs
16	Yunji	0.856	0.863	0.992	drs
17	Weimob	0.473	0.559	0.846	drs
18	Youzan	0.305	0.329	0.927	drs
19	Ellassay	0.721	0.845	0.854	drs
20	Ruhnn	0.432	0.614	0.703	irs
21	Yujiahui	0.866	0.868	0.997	drs
22	111.com	0.804	0.881	0.913	drs
23	Babytree	0.525	1	0.525	irs
24	Secoo	1	1	1	-
25	Mogu	0.571	0.591	0.966	drs
26	Jumei	0.727	0.728	0.998	drs
27	Tuanche	1	1	1	-
	Mean	0.721	0.846	0.860	
	B2B				
28	Shanghai Ganglian	1	1	1	-
29	GlobalTop	0.971	1	0.971	drs
30	United Information	0.876	0.877	1	-
31	Lianluo Interactive	1	1	1	-
32	Zall Smart	0.977	0.978	0.999	irs
33	Tianze Information	0.590	0.61	0.967	drs
34	Guanfu	0.827	0.828	0.999	irs
35	Netsun	0.309	0.356	0.869	irs
36	Focus	0.536	0.537	0.998	irs
37	Huading	0.745	0.804	0.927	drs
38	Molbase	1	1	1	-
39	Guangbo	0.613	0.626	0.979	drs
40	HC Group	0.746	0.759	0.983	drs
41	Cogobuy	0.721	0.763	0.945	irs
42	Europol	0.358	1	0.358	irs
43	LightIn TheBox	1	1	1	-
44	DX.COM	1	1	1	-
	Mean	0.781	0.832	0.941	
	P2C				
45	Meituan	0.559	0.996	0.561	drs
46	Ctrip	0.491	1	0.491	drs
47	Alibaba Health	1	1	1	-
48	58.com	0.999	1	0.999	drs
49	Luckin	0.471	0.615	0.766	drs
50	Ping An Health Cloud	0.432	0.463	0.934	drs
51	51job	0.435	0.498	0.874	drs
52	Genshuixue	0.273	0.278	0.982	drs
53	Alibaba Picture	0.254	0.262	0.967	irs
54	Tongcheng-Elong	0.572	0.663	0.863	drs
55	Koolearn	0.342	0.368	0.928	drs
56	Maoyan	0.549	0.549	1	-
57	Youdao	0.676	0.755	0.896	drs
58	So-Young	0.639	0.84	0.761	irs
59	Fangdd	0.219	0.22	0.995	irs
60	Q&K	0.581	0.633	0.916	drs
61	Qeeka	0.530	0.555	0.955	irs
62	Tuniu	0.412	0.537	0.768	drs
63	Leju	1	1	1	-
64	Soufun	0.774	1	0.774	irs
65	51Talk	0.855	0.894	0.957	drs
	Mean	0.574	0.673	0.876	
	Total mean	0.689	0.786	0.886	

**Note:** CRSTE = technical efficiency based on the CRS data envelopment analysis (DEA) approach’s = technical efficiency based on the VRS DEA approach; Scale = scale efficiency (CRSTE/VRSTE).

After eliminating the effect of scale efficiency, we examined the change trend of pure technical efficiency of all types of e-commerce firms. The pure technical efficiency of B2B firms was found to be higher than that of B2B and P2C firms. Furthermore, the pure technical efficiency of B2C and P2C firms initially tended to decrease and subsequently increase, whereas that of B2B firms initially tended to increase and then decrease; the same results were observed for the trend of technical efficiency. During 2015 and 2019, the pure technical efficiency of B2C and P2C firms declined by 2.7% and 0.8%, respectively. P2C firms exhibited the greatest decline of 13.5% in pure technical efficiency.

Between 2015 and 2019, the scale efficiency of firms tended to increase initially and then decrease, indicating that Chinese e-commerce firms focused on technical progress, rather than simply scaling up, to improve their operating efficiency. B2B firms showed the highest scale efficiency, followed by P2C and B2C firms. This situation conformed to the actual operating conditions; that is, B2B firms usually are larger in scale, whereas B2C firms usually are smaller in scale. Between 2015 and 2019, the scale efficiency of B2C, B2B, and P2C firms decreased by 4.6%, 4.2%, and 5.3%, respectively.

### 4.2 Second-stage SFA: Environmental factor analysis and input variable adjustment

In this study, we chose the input slack obtained from the estimated results of the first-stage DEA model as dependent factors and the five environmental variables as independent factors. Frontier 4.1 software and maximum likelihood estimation were used to investigate the effect of environmental variables on technical efficiency. As shown in Table 3, the five environmental variables reached significance at the 1% level, indicating that the choice of environmental variables was reasonable. Because the environmental factors exerted a significant effect on the input slack, it was necessary to eliminate the effect of these factors on input variables and to adjust input variables. A positive estimated coefficient indicated that an increase in the environmental variables would cause an increase in the input slack, whereas a negative estimated coefficient indicated that an increase in the environmental variables would cause a decrease in the input slack. According to the regression coefficients, we obtained the following findings [9]:

1. Years of incorporation exerted a positive effect on total assets and operating cost slack but a negative effect on the number of employees. The results indicated that the longer the years of incorporation, the greater the waste of total assets and operating costs, which was not harmful to the improvement of operating efficiency.
2. Ownership concentration exerted a positive effect on operating cost and total asset slack. The results indicated that higher ownership concentration resulted in the substantial waste of operating costs and total assets. The average proportion of shares held by the first shareholders of 65 e-commerce firms was 35.66%; among the firms, Youzan had the highest proportion of 97%. Thus, it was easy to produce the “internal control” phenomenon and restrict the improvement of operating efficiency.
3. The equity restriction ratio exerted a positive effect on the number of employees and operating cost slack, indicating that a high equity restriction ratio enhanced the input waste of the number of employees and operating costs. This was because a higher ownership concentration was harmful to the effect of the equity restriction ratio.
4. The Internet development index exerted a negative effect on operating cost and total asset slack, indicating that improvements in regional Internet development level could optimize resource allocation and reduce the waste of operating cost and total assets.
5. Degree of regional openness exerted a positive effect on every input slack, indicating that the greater the degree of regional openness, the lower the input slack. This finding demonstrates that firms in the open region made full use of regional advantages, and regional openness promoted the improvement of operating efficiency.

**Table 3:** Second-stage stochastic frontier analysis regression results.

Variables	Number of staffs	Total assents	Operating cost
Constant (β0)	-362.76*** (-221.31)	380.44*** (196.19)	597.32*** (462.77)
Years of incorporation (β1)	112.13*** (217.43)	-471.31*** (-342.18)	17.54*** (111.33)
Ownership concentration (β2)	-92.11*** (-200.76)	12.53*** (96.82)	464.98*** (804.64)
Equity restriction ratio (β3)	9.26*** (46.58)	3.83*** (102.33)	10.14*** (58.83)
Internet development index (β4)	60.33*** (22.31)	-582.43*** (-298.96)	-154.56*** (-128.38)
Regional openness degree (β5)	1.32*** (6.56)	2.30*** (1.53)	3.61*** (4.89)
$\sigma^{2}=\sigma_{v}^{2}+\sigma_{\mu }^{2}$	877299.00*** (877299.00)	33719.00*** (33719.00)	58051.00*** (58051.00)
$\gamma =\sigma_{\mu }^{2}+\sigma^{2}$	1.00*** (843513.00)	1.00*** (83668.66)	1.00*** (576.22)
Log likelihood function	-760.46	-579.71	-522.53

Note:

(***), (**), and (*) respectively represent significance at the 1%, 5%, and 10% level.

In sum, the environmental factors exerted a significant effect on the input slack of e-commerce listed firms, and these factors had different effect directions and strengths. The efficiency estimation of original data without elimination of the effect of environmental factors resulted in a lack of authenticity and scientific. Hence, it was necessary to adjust the original input variables by using the estimation coefficient from the second-stage SFA results to eliminate the effect of random factors and analyze firms under the same outside environment [43].

### 4.3 Third-stage DEA: BCC model analysis based on input after adjustment

We again used DEAP2.1 to estimate the BCC model by using the adjusted input and output and obtain the authentic efficiency of 65 e-commerce firms. Subsequently, we compared these results with those obtained in the first and third stages (Table 4).

**Table 4:** Comparison of technical efficiency after adjustment for the input variables of 65 e-commerce firms in 2019.

No.	DMU	crste	vrste	scale	return to scale
	B2C				
1	Alibaba	0.492	1	0.492	drs
2	Jingdong	0.820	1	0.820	drs
3	Pinduoduo	0.711	1	0.711	drs
4	Xiaomi Group	0.722	1	0.722	drs
5	Sunning	0.67	1	0.670	drs
6	Vipshop	0.809	1	0.809	drs
7	Nanji	0.983	1	0.983	irs
8	Three squirrels	0.995	1	0.995	drs
9	Lexin	0.975	1	0.975	drs
10	Baozun	0.994	1	0.994	drs
11	Onechance	0.990	1	0.990	irs
12	Gome	0.703	1	0.703	drs
13	Qudian	1	1	1	-
14	Bea	0.943	1	0.943	drs
15	Zhidemai	0.807	0.991	0.814	irs
16	Yunji	0.999	1	0.999	drs
17	Weimob	1	1	1	-
18	Youzan	0.856	1	0.856	irs
19	Ellassay	0.997	1	0.997	drs
20	Ruhnn	0.478	1	0.478	irs
21	Yujiahui	0.999	1	0.999	drs
22	111.com	0.995	1	0.995	drs
23	Babytree	0.525	1	0.525	irs
24	Secoo	1	1	1	-
25	Mogu	0.986	1	0.986	drs
26	Jumei	0.998	1	0.998	irs
27	Tuanche	1	1	1	-
	Mean				
	B2B				
28	Shanghai Ganglian	1	1	1	-
29	GlobalTop	0.971	1	0.971	drs
30	United Information	0.876	0.877	1	-
31	Lianluo Interactive	1	1	1	-
32	Zall Smart	0.999	1	0.999	irs
33	Tianze Information	1	1	1	-
34	Guanfu	0.996	1	0.996	irs
35	Netsun	0.469	1	0.469	irs
36	Focus	0.801	1	0.801	irs
37	Huading	0.972	1	0.972	drs
38	Molbase	1	1	1	-
39	Guangbo	0.997	1	0.997	drs
40	HC Group	0.987	1	0.987	drs
41	Cogobuy	0.918	1	0.918	irs
42	Europol	0.358	1	0.358	irs
43	LightIn TheBox	1	1	1	-
44	DX.COM	1	1	1	-
	Mean				
	P2C				
45	Meituan	0.569	1	0.569	drs
46	Ctri	0.491	1	0.491	drs
47	Alibaba Health	1	1	1	-
48	58.com	0.999	1	0.999	drs
49	Luckin	0.999	1	0.999	drs
50	Ping A Health Cloud	0.983	1	0.983	drs
51	51job	1	1	1	-
52	Genshuixue	0.680	1	0.680	irs
53	Alibaba Picture	0.712	1	0.712	irs
54	Tongcheng-Elong	1	1	1	-
55	Koolearn	0.619	1	0.619	irs
56	Maoyan	0.964	1	0.964	irs
57	Youdao	0.989	1	0.989	drs
58	So-Young	0.690	1	0.691	irs
59	Fangdd	0.657	1	0.657	irs
60	Q&K	1	1	1	-
61	Qeeka	0.747	1	0.747	irs
62	Tuniu	0.986	1	0.986	drs
63	Leju	1	1	1	-
64	Soufun	0.774	1	0.774	irs
65	51Talk	1	1	1	-
	Mean	0.872	0.998	0.874	
	Total mean				

**Note:** CRSTE = technical efficiency based on the CRS data envelopment analysis (DEA) approach. VRSTE = technical efficiency based on the VRS DEA approach. Scale = scale efficiency (CRSTE/VRSTE).

With regard to overall technical efficiency, after comparing the efficiency score of the two stages, we found that the average operating efficiency decreased from 0.689 before adjustment to 0.872 after adjustment, with an improvement of 27%. The average pure technical efficiency increased from 0.786 before adjustment to 0.998 after adjustment, with an improvement of 27%. However, the average scale efficiency declined slightly from 0.886 to 0.874, with a decrease of 1.35%. After eliminating the effect of environmental factors and random error, we observed significant increases in operating efficiency and pure technical efficiency but a slight decrease in scale efficiency. These findings indicated that the management and resource allocation levels were above average, and the unideal external environmental conditions and more random errors resulted in ineffective pure technical efficiency in the first stage. However, ineffective scale efficiency resulted in ineffective operating efficiency in the third stage.

In particular, 98% of firms showed a significant improvement in operating efficiency. The operating efficiency of 9 B2C firms was constant, and that of the other 18 firms increased slightly. However, one firm (Mogu) exhibited a decline in operating efficiency, and Youzan demonstrated the largest increase of 180.7% in operating efficiency. Furthermore, 6 B2B firms demonstrated constant operating efficiency, and the other 11 firms showed a significant increase in operating efficiency; of these 11 firms, Tianze Information demonstrated the largest increase of 69.5%. Furthermore, 6 P2C companies had constant operating efficiency, and the other 15 firms showed an increase in operating efficiency; of these 15 firms, Alibaba Picture demonstrated the largest increase of 180.3% in operating efficiency.

A total of 10 B2C firms demonstrated constant pure technical efficiency, and the other 17 firms showed a significant decrease in pure technical efficiency; among the firms, Youzan showed the largest increase of 203%. Furthermore, 7 B2B firms demonstrated constant pure technical efficiency, and the other 9 firms showed a significant increase in pure technical efficiency; among these firms, Netsun revealed the largest increase of 180.9%. One firm, namely United Information, demonstrated a slight decline of 0.11% in pure technical efficiency. In addition, 5 P2C firms showed constant pure technical efficiency, and the other 16 firms revealed a significant increase in pure technical efficiency; among these firms, Fangdd demonstrated the largest increase of 354.6%.

A total of 9 B2C firms had constant scale efficiency, and the other 13 firms exhibited a significant increase in scale efficiency; among these firms, Weimob revealed the largest increase of 18.3%. However, five firms showed a decline in scale efficiency, of which Ruhnn had the largest decrease of 32%. Furthermore, 5 P2C firms had constant scale efficiency, and the other 9 firms showed a significant increase in scale efficiency; among these firms, Luckin demonstrated the greatest increase of 30.4%. However, seven firms showed a significant decline, among which Fangdd had the largest decline of 34%.

We calculated the operating efficiency of different types of e-commerce firms, and the results are listed in Table 5. Before and after the adjustment of input variables, the ranking of the three types of e-commerce firms was constant. B2B firms had the highest efficiency, followed by B2C and P2C firms. When combined with the practical situation, we observed that mature B2B platforms attracted numerous foreign trade firms to enter because of their complete operation mode, low costs, and low barriers to entry; thus, B2B firms were chosen as the most common mode by most small and medium enterprises. B2C platform firms conducted cross-border trade through third-party platforms. This enabled them to save substantial costs in terms of platform construction and operation management. Thus, B2C firms focused on the development of their own products by making use of the traffic of third-party platforms. Some B2C firms had their own websites to perform online transactions, and most of them were industry leaders with extensive experience. The self-built platform had a high degree of compatibility with enterprise products and had considerable advantages in e-commerce. Most P2C firms provided services in logistics, finance, and storehouse and had different operation modes; thus, their development level was uneven. Moreover, because e-commerce service firms require a substantial amount of infrastructure investment, their development was backward.

**Table 5:** Comparison of technical efficiency before and after adjustment of the input variables of three types of e-commerce firms in 2019.

Type	crste			vrste			scale		
	before	after	Improvement percentage	before	After	Improvement percentage	before	after	Improvement percentage
B2C	0.721	0.868	20.38%	0.850	1	17.67%	0.855	0.869	1.55%
B2B	0.782	0.903	15.45%	0.835	0.993	18.89%	0.938	0.948	1.00%
P2C	0.574	0.872	51.80%	0.675	0.998	47.88%	0.872	0.874	0.17%
Total	0.689	0.872	26.56%	0.786	0.998	26.97%	0.886	0.874	-1.35%

### 4.4 DEA–Malmquist TFP index

The BCC model in DEA was used to statically analyze the panel data of enterprises. However, because the operation and management of enterprises is a self-sustaining process, it should be evaluated dynamically. The Malmquist index model overcomes the deficiency of the static mode. This study analyzed the TFP index and its component as well as examined the change value by using the panel data of 65 e-commerce firms from 2015 to 2019. This study aimed to determine the change trend of e-commerce companies in different years and explore factors that affect operating performance [35].

TFP index for the period from 2018 to 2019 was >1 during the period from 2015 to 2019, indicating that the operating efficiency of Chinese e-commerce firms began improving from 2018. In terms of the average MPI, the average technical efficiency change showed a decline of 13%, and the average technical progress change index showed an improvement of 21%, resulting in an improvement of 5% in the average MPI. These findings indicated that most e-commerce firms actively introduced advanced technology during 2015–2019 to promote technical productivity. The increase in technical progress has been the key factor responsible for the growth of the MPI, bringing about favorable development opportunities for the whole industry.

**Fig 2:**
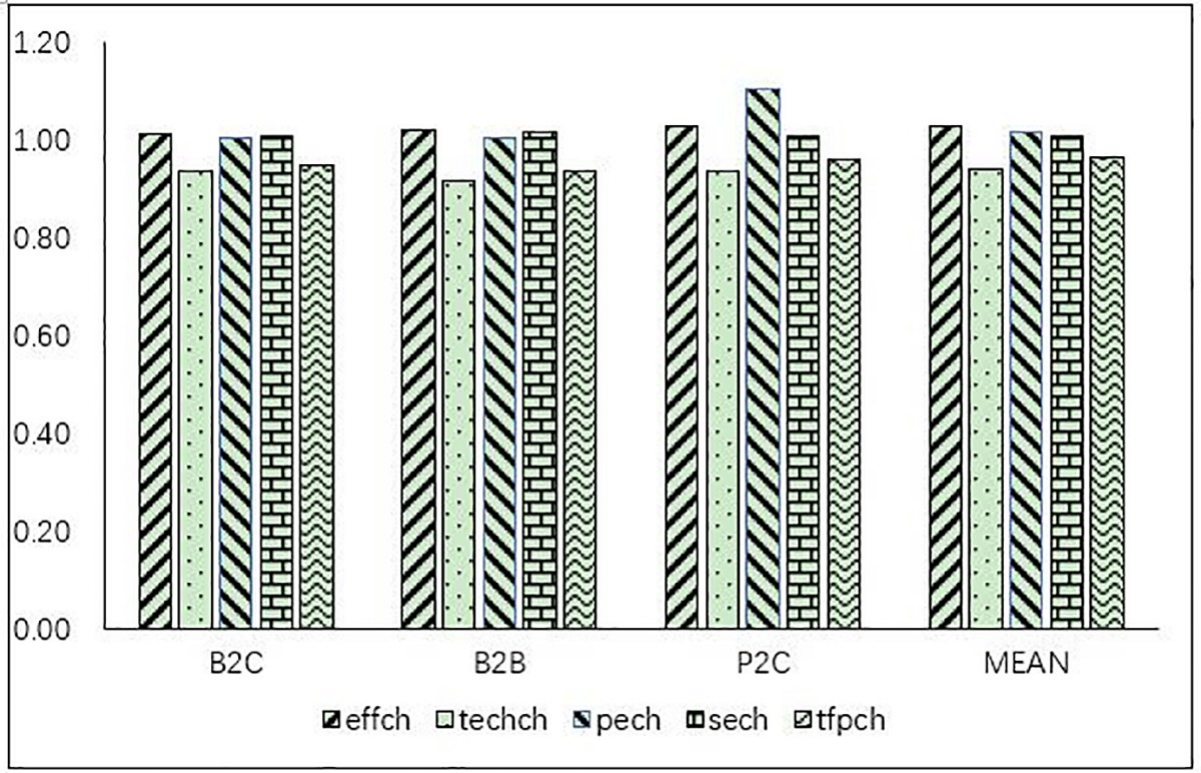
Summary of the average Malmquist index of different types of e-commerce firms.

Pure technical efficiency and scale efficiency initially tended to decrease and then increase. In general, pure technical efficiency and scale efficiency declined by 5% and 9%, respectively. Because 2015 was a major year in terms of e-commerce development, the e-commerce industry developed rapidly with the double positive factors of policies and market. Thus, e-commerce firms should seize the core competency and increase their scale to promote development and growth until they adopt advanced technology and make a breakthrough in innovation. As displayed in Table 6 and Fig 2, we arranged the 65 e-commerce firms in descending order of their MPI. On the basis of the findings, the following conclusions can be drawn:

1. The average MPI was 0.967 in 2019, and only seven B2C firms, including Weimob, Tuanche, and Bear, had an MPI of >1, accounting for 26% of all B2C firms. Only four B2B firms had an MPI of >1, accounting for 24% of all B2B firms. Eleven P2C firms had an MPI of >1, accounting for 52% of all P2C firms. These findings indicated that the overall operating efficiency of the Chinese e-commerce industry was increasing. Being a new business form in international trade associated with the development of the Internet, e-commerce has enormous growth potential under the Belt and Road Initiative and for the deep integration of global economy and trade. Simultaneously, the promulgation of favorable policies, the progress of Internet technology, and the upgrade of consumer purchasing power have provided a favorable development environment for the e-commerce industry.
2. Regarding the decomposition of the MPI, most firms had a technical efficiency change index (EFFCH) of >1, among which B2C firms accounted for 70% and Weimob had the highest EFFCH index. Furthermore, 71% of the total B2B firms had an EFFCH index of >1, and Europol had the highest EFFCH index. In addition, 76% of the total P2C firms had an EFFCH index of >1, and Luckin had the highest EFFCH index. These findings indicated that most firms had increasing returns on investment, and their technical efficiency was increasing. Only six B2C firms, two B2B firms, and six P2C firms had a technical progress change index (TECHCH) of >1, and the average TECHCH index was 0.941. However, the overall technical progress change index was increasing. In these 5 years, technology in the e-commerce industry was progressive and exerted a substantial promoting effect.

**Table 6:** Summary of the Malmquist total factor productivity index of different types of firms during 2015–2019 (ranked by TFPCH).

No.	DMU	effch	techch	pech	sech	tfpch
	B2C					
1	Weimob	1.206	1.144	1.156	1.043	1.380
2	Tuanche	1	1.108	1	1	1.108
3	Bear	1.037	1.044	1.071	0.968	1.082
4	Jumei	1.150	0.925	1.137	1.012	1.064
5	Gome	1.124	0.930	1.046	1.075	1.045
6	Youzan	1.032	1.005	1.026	1.005	1.037
7	Zhidemai	1.024	0.976	1.061	0.965	1
8	Onechance	1.011	0.988	1.001	1.010	0.999
9	Three squirrels	0.999	1.001	1.012	0.987	0.999
10	Alibaba	1.180	0.842	1	1.180	0.994
11	Sunning	1.083	0.914	1	1.083	0.991
12	Vipshop	1.050	0.940	1	1.050	0.987
13	Yunji	1.027	0.944	1.026	1	0.969
14	Yujiahui	1.037	0.931	1.036	1.001	0.965
15	Secoo	1.049	0.917	1.047	1.002	0.962
16	Baozun	0.989	0.935	0.985	1.004	0.925
17	Jingdong	1.002	0.919	1	1.002	0.921
18	Babytree	1.093	0.838	0.988	1.107	0.916
19	111.com	0.937	0.949	0.912	1.027	0.889
20	Nanji	1.029	0.861	1.026	1.004	0.886
21	Xiaomi Group	1.053	0.840	1	1.053	0.884
22	Ruhnn	0.972	0.901	0.976	0.996	0.876
23	Lexin	0.958	0.895	0.937	1.022	0.857
24	Ellassay	0.729	1.156	1.130	0.645	0.842
25	Mogu	1	0.794	1	1	0.794
26	Pinduoduo	0.779	0.919	0.772	1.010	0.716
27	Qudian	0.829	0.708	0.844	0.983	0.587
	**Mean**	**1.014**	**0.938**	**1.007**	**1.009**	**0.951**
	B2B					
28	Europol	1.293	0.920	1	1.293	1.190
29	LightIn TheBox	1	1.072	1	1	1.072
30	Netsun	1.207	0.869	1.212	0.996	1.049
31	DX.COM	1	1.015	1	1	1.015
32	Guangbo	1.008	0.990	1.005	1.002	0.997
33	Cogobuy	1.085	0.909	1.070	1.014	0.986
34	United Information	0.997	0.983	1.012	0.985	0.980
35	HC Group	1.076	0.907	1.067	1.008	0.976
36	Molbase	1	0.932	1	1	0.932
37	Zall Smart	1.007	0.899	1.008	1	0.906
38	Lianluo Interactive	0.953	0.922	0.955	0.997	0.878
39	Focus	1.010	0.881	1.015	0.996	0.876
40	Guanfu	0.975	0.890	0.980	0.995	0.868
41	Shanghai Ganglian	1	0.826	1	1	0.826
42	Huading	0.883	0.923	0.855	1.033	0.815
43	Tianze Information	0.867	0.889	0.876	0.990	0.771
44	GlobalTop	1	0.767	1	1	0.767
	**Mean**	**1.021**	**0.917**	**1.003**	**1.018**	**0.936**
	P2C					
45	Luckin	1.207	1.247	1.129	1.069	1.505
46	Genshuixue	1.253	1.128	1.270	0.986	1.414
47	Fangdd	1.379	0.901	1.378	1.001	1.242
48	Meituan	1.157	1.015	1.001	1.155	1.174
49	Qeeka	1.172	1	1.159	1.011	1.172
50	Youdao	1.064	1.043	1.061	1.003	1.110
51	51Talk	1.040	1.055	1.028	1.011	1.097
52	Maoyan	1.161	0.938	1.161	1	1.089
53	Tuniu	1.152	0.909	1.101	1.047	1.047
54	Koolearn	1.064	0.950	1.073	0.992	1.011
55	Ping An Health Cloud	1.064	0.944	1.110	0.958	1.004
56	51job	1.123	0.866	1.174	0.956	0.973
57	Leju	1	0.971	1	1	0.971
58	Q&K	0.985	0.985	0.972	1.013	0.969
59	So-Young	0.979	0.980	0.922	1.062	0.959
60	58.com	1	0.944	1	1	0.944
61	Tongcheng-Elong	0.973	0.953	1.022	0.953	0.928
62	Soufun	0.904	0.959	0.852	1.062	0.867
63	Ctrip	1.017	0.846	0.967	1.052	0.861
64	Alibaba Picture	1.125	0.755	1.117	1.007	0.850
65	Alibaba Health	0.687	0.927	0.690	0.997	0.637
	**Mean**	**1.028**	**0.937**	**1.107**	**1.011**	**0.963**
	Total mean	1.028	0.941	1.018	1.011	0.967

Note: EFFCH: technical efficiency change; TECHCH: technological change; PECH: pure technical efficiency change; SECH: scale efficiency change; TFPCH: Malmquist TFP index.

All Malmquist index averages are geometric means.

Most firms (20 B2C, 13 B2B, and 11 P2C firms) had a pure technical efficiency change (PECH) index of >1 (accounting for 74%, 76%, and 76%, respectively). These findings indicated that most of the e-commerce firms focused on technical innovation and made some progress.

Overall, 22 B2C, 11 B2B, and 15 P2C firms had a scale efficiency change index (SECH) index of >1, accounting for 81%, 65%, and 71%, respectively. These firms had a SECH index of >1 because they expanded their scale and increased their inputs, resulting in decreased returns to scale. Furthermore, when we combined the PECH and SECH, we observed that when one of them was effective, then technical efficiency was directly associated with the other index. When both of them were effective, then technical efficiency was also effective. When neither of them was effective, technical efficiency was also not effective.

## 5. Conclusions and recommendation

This study calculated the technical efficiency and MPI of 65 e-commerce firms in China from 2015 to 2019. The operating efficiency and MPI of e-commerce firms have rarely been assessed in China. This study applied a nonparametric three-stage DEA method and MPI to estimate the technical efficiency and total productivity index and its decomposition by classifying e-commerce firms into B2C, B2B, and P2C firms. This study focused on the effect of outside environmental factors on the input slack and defined environmental factors to examine their effects on different inputs. In addition, this paper proposes a new adjustment format on the basis of the estimation of environmental factors in the traditional SFA mode. We drew the following conclusions:

1. According the analysis results of environmental variables, we found that the operating efficiency was considerably affected by environmental factors. An increase in years of incorporation increased the waste of total assets and operating cost. Increased ownership concentration restricted the improvement of operating efficiency and was harmful to the effect of the equity restriction ratio. Improvements in the regional Internet development level could reduce the slack of operating cost and total assets, and a high degree of regional openness could reduce the waste of input and improve technical efficiency.
2. The analysis results of technical efficiency revealed that most e-commerce firms exhibited an improvement in operating efficiency and pure technical efficiency, and scale efficiency slightly declined after the effect of environmental and random factors was eliminated. The findings indicate that the management and resource allocation level was above average. The unideal external environmental condition and more random errors were the main reasons for the low pure technical efficiency in the first stage; however, low scale efficiency caused low operating efficiency in the third stage.
3. From the analysis results of different types of e-commerce firms, we observed that B2B firms had the highest technical efficiency, followed by B2C and P2C firms. However, firms should confirm the improvement direction of operating efficiency according to the condition of pure technical efficiency and scale efficiency. In the case of low scale efficiency, firms should expand their scale and focus on scale operation. In the case of low pure technical efficiency, firms should focus on increasing the enterprise management and decision ability. In firms with both ineffective pure technical efficiency and ineffective scale efficiency, the management level should be optimized and the scale should be expanded.
4. After dynamically analyzing the total productivity index by using the DEA–Malmquist method, we found that technical progress efficiency (i.e., the MPI) was the key factor for improving productivity. Although the technical efficiency during 2015–2019 was ineffective, the technical progress efficiency was effective, resulting in an effective MPI. At the same time, both pure technical efficiency and scale efficiency were found to be significant factors ensuring effective technical efficiency. However, most e-commerce firms included in this study had relatively low scale efficiency, which is not conducive for achieving technical efficiency. Hence, e-commerce firms should improve their scale efficiency to achieve sustainable development.

On the basis of these findings, we provide some suggestions for e-commerce firms and the whole industry [44]. First, firms should focus on technological innovation. From 2015, China’s e-commerce industry entered the era of online transformation of the whole industrial chain process. In addition, 5G is expected to soon be available, and new technologies, such as artificial intelligence and virtual reality, have penetrated the industry. In this context, e-commerce firms should strengthen technical innovation and change the original operating concept of emphasizing profit over technology. They should increase investments and introduce advanced software and hardware equipment by using cloud computing and big data to increase data-handling capacity.

Second, firms should improve cross-border logistics system construction. Currently, many firms encounter high logistics costs, long delivery times, and difficulty in returning or exchanging goods. Thus, firms should improve their own logistics system construction. To solve these problems, firms can cooperate with other firms by leasing out, contracting, or buying a share to cobuild overseas storehouses to increase their delivery efficiency. However, in the context of intelligent logistics, firms can cooperate with each other to cobuild logistics information-sharing platforms to construct a whole solution to increase the risk-handling capacity.Finally, firms should expand their scale appropriately. Many e-commerce firms are small firms or microbusinesses. They cannot succeed without expanding their scale. Hence, firms should increase their investments and stock-keeping units to expand their scale and promote the development of the e-commerce industry. Limitations of this paper are as follows: the sample size is not large enough to show the overview of the e-commerce industry. The variables are not scientific enough to illustrate the actual operation condition of e-commerce firms. In the further research, we will calculate the operation efficiency of e-commerce companies with more sample firms and other precise methods or we will study on the operation efficiency of cross-border e-commerce companies in the world.

## Supporting information

S1 Dataset (XLSX)
